# ASSOCIATION BETWEEN ORAL HYGIENE BEHAVIORS AND COGNITIVE FUNCTION

**DOI:** 10.1093/geroni/igae098.1630

**Published:** 2024-12-31

**Authors:** Zheng Zhu, Zhongfang Yang, Xiang Qi, Weiyu Mao, Yaolin Pei, Bei Wu

**Affiliations:** New York University, New York, New York, United States; Fudan University, Shanghai, Shanghai, China (People’s Republic); New York University, New York, New York, United States; University of Nevada, Reno, Reno, Nevada, United States; University of Texas at Austin, Texas, United States; New York University, New York, New York, United States

## Abstract

**Background:**

Improving oral hygiene behaviors may offer a novel approach to delay cognitive decline; however, there is limited data available on its effectiveness in affecting cognitive function. This review aims to examine whether oral hygiene behaviors would be an effective strategy to improve cognitive function. Method: A search of 11 literature databases were searched from inception through January 6, 2024. Interventional trials or cohort studies aiming to examine the effectiveness of oral hygiene behaviors in affecting cognitive function in adults were included. Oral hygiene behaviors included, but were not limited to, toothbrushing, mouthwashing, flossing, using toothpicks, and cleaning dentures. The primary outcome was the changes in global cognitive function. The meta-analysis was carried out using random-effects models.

**Results:**

A total of 8 studies met inclusion criteria, including 261,772 participants. Toothbrushing was associated with a significantly higher level of global cognitive function (Mean Difference [MD], 0.65 [95% CI, 0.19-1.10]; I2=2%). There was no significant difference between the effectiveness of toothbrushing performed by professionals (MD, 0.93 [95% CI, 0.20-1.67]; I2=6%) and caregivers (MD, 1.01 [95% CI, 0.39 to 1.62]; I2=3%) (P > 0.05). Other oral hygiene behaviors including dental flossing, using mouthwash, using toothpicks, and cleaning dentures, were not associated with the risk of dementia.

**Discussion:**

Regular toothbrushing is not only linked to improvements in global cognitive function and a reduced risk of dementia but also improve specific cognitive function subdomains such as visual attention and task-switching ability.

